# Neurological heterotopic ossification: novel mechanisms, prognostic biomarkers and prophylactic therapies

**DOI:** 10.1038/s41413-020-00119-9

**Published:** 2020-12-09

**Authors:** Ker Rui Wong, Richelle Mychasiuk, Terence J. O’Brien, Sandy R. Shultz, Stuart J. McDonald, Rhys D. Brady

**Affiliations:** 1 Department of Neuroscience, Central Clinical School, Monash University, Melbourne, VIC Australia; 2 Department of Medicine, Royal Melbourne Hospital, The University of Melbourne, Parkville, VIC Australia; 3 Department of Physiology, Anatomy and Microbiology, School of Life Sciences, La Trobe University, Bundoora, VIC Australia

**Keywords:** Diseases, Bone

## Abstract

Neurological heterotopic ossification (NHO) is a debilitating condition where bone forms in soft tissue, such as muscle surrounding the hip and knee, following an injury to the brain or spinal cord. This abnormal formation of bone can result in nerve impingement, pain, contractures and impaired movement. Patients are often diagnosed with NHO after the bone tissue has completely mineralised, leaving invasive surgical resection the only remaining treatment option. Surgical resection of NHO creates potential for added complications, particularly in patients with concomitant injury to the central nervous system (CNS). Although recent work has begun to shed light on the physiological mechanisms involved in NHO, there remains a significant knowledge gap related to the prognostic biomarkers and prophylactic treatments which are necessary to prevent NHO and optimise patient outcomes. This article reviews the current understanding pertaining to NHO epidemiology, pathobiology, biomarkers and treatment options. In particular, we focus on how concomitant CNS injury may drive ectopic bone formation and discuss considerations for treating polytrauma patients with NHO. We conclude that understanding of the pathogenesis of NHO is rapidly advancing, and as such, there is the strong potential for future research to unearth methods capable of identifying patients likely to develop NHO, and targeted treatments to prevent its manifestation.

## Introduction

Heterotopic ossification (HO) is the pathological formation of bone in muscles and surrounding joints. The ramifications of this ectopic bone formation in soft tissue include swelling, pain, nerve entrapment, contractures, and in some cases, limited range of movement due to bone fusion in the affected area (i.e. ankylosis).^1^ Although various forms of hereditary and acquired HO exist, HO that occurs following a neurological insult (i.e. neurological HO; NHO) is of increasing clinical concern due to its rising prevalence in combat and civilian populations.^2^ Neurological heterotopic ossification (NHO) is particularly common when a neurological insult, such as a traumatic brain injury (TBI) or spinal cord injury (SCI), occurs in the presence of concomitant peripheral injuries (e.g. bone fractures, muscle injuries). Non-surgical interventions include analgesia,^3^ rest,^4^ and nerve blockers;^5^ however, the only cure is surgical excision.^6,7^ Unfortunately, invasive surgical excision can only occur once the lesion has mineralised, and reoccurs in ~6% of patients.^8^ Consequently, there is an urgent need to develop prophylactic interventions that can prevent HO and are still appropriate for patients who have sustained severe trauma to the periphery and central nervous system (CNS).^9^ To date, the development of prophylaxes has been hindered by a limited understanding of how ectopic bone formation is triggered in response to CNS and musculoskeletal trauma. Nonetheless, there has been a recent rise in the number of clinical and pre-clinical studies designed to unearth the cellular and molecular mechanisms of NHO. This article will first review the literature regarding NHO epidemiology, the pathophysiology of CNS injuries, and how they may promote bone formation, mechanisms of endochondral bone formation before presenting new research avenues towards identifying novel mechanisms of NHO, and the limited NHO treatment options. As the literature specific to NHO is relatively limited, we sometimes draw from the broader HO literature and make inferences where relevant.

## Epidemiology and risk factors for NHO

It has been reported that at least 10%–20% of patients with a TBI and simultaneous peripheral musculoskeletal injuries (i.e. polytrauma involving substantial muscle injuries and bone fractures) develop NHO.^10–13^ Following combined SCI and polytrauma, ~15%–30% of patients develop NHO.^10–12^ This ectopic bone has a tendency to form predominantly in muscle surrounding the hip, although it also frequently affects other joints such as the knee, elbow, and shoulder (see Table 1).^10–12^ Notably, when NHO is formed posteriorly at the hip, it often entraps the sciatic nerve, resulting in neurological pain and muscle weakness, impaired movement, and difficulty performing everyday tasks (e.g. standing, sitting and getting dressed).^10,14^ Most studies suggest that NHO prevalence is significantly higher in males than females, which has been attributed to the increased number of males that experience a TBI or SCI.^15^ However, a recent study employing a mouse model of HO (featuring a dermal burn and Achilles tenotomy) reported that male mice formed ~30% more ectopic bone when compared to female mice, possibly due to increased insulin like growth factor-1 and bone morphogenetic protein (BMP) signalling in males.^16^ Therefore, males may be predisposed to having increased risk for the development of HO/NHO.

**Table 1:** Incidence, demographics and locations of NHO reported across the literature

Author	Description (*total number of* patients, age range, mean age, gender, *number of* patients with NHO)	Type of injury/s	Location of HO and imaging modality used
Reznik et al.^165^	262 TBI patients, 226 M, 36 F TBI-NHO patients: 10, mean: 39.6 years, 10 M 151 SCI patients, 128 M, 23 F 16 SCI-NHO patients, mean: 31.4 years, 13 M, 3 F	TBI and SCI	Hip: 17 lesions, shoulder: 1 lesion, elbow: 3 lesions, knee: 5 lesions Bone scintigraphy
Dizdar et al.^20^	151 TBI patients, 126 M, 25 F 56 NHO patients, mean: 34.6 years, 48 M, 8 F	TBI	Hip: 41 patients, shoulder: 11 patients, elbow: 20 patients, knee: 25 patients, ankle: 2 patients Imaging modality: not reported (NR)
Van Kampen et al.^22^	176 TBI patients, 75 M, 22 F 13 TBI-NHO patients, mean: 34.65 years, 10 M, 3 F 79 excluded GCS <8. Coma duration in NHO group: 15 days Coma duration in non-NHO group: 4.18 days Mechanical ventilation: 17.23 days Immobilisation days: 13.46	TBI Concomitant bone FX: 8/13 patients	Hip: 3 lesions, shoulder: 2 lesions, thigh: 2 lesions, elbow: 4 lesions, knee: 5 lesions, femur: 1 lesion, ankle: 1 lesion, iliopsoas muscle: 1 lesion Imaging modality: NR
Rigaux et al.^166^	31 TBI patients, 31 M 12 TBI-NHO patients, 33 years,12 M GCS: ≥8 3 months-post-injury	TBI	NR X-ray, bone scintigraphy
Hurvitz et al.^167^	90 TBI patients, mean: 11.9 years, 67 M, 23 F 13 TBI-NHO patients, 9 M, 4 F 35 patients TBI + extremity FX 30 patients TBI + skull FX	TBI	Hip: 4 lesions, shoulder: 3, femur: 3, elbow: 3, knee: 4 lesions, forearm: 2, ischium: 2 X-ray, bone scintigraphy
Hendricks et al.^168^	76 TBI patients, 16–84 years, mean: 36.67 years, 47 M 29 F 9 TBI-NHO patients GCS 3: 9 Coma duration of 76 patients: 10.03 (1–61 days) Mechanical ventilation: 8.95 (0–52 days) Diffuse axonal injury: 7 patients	TBI Concomitant bone FX: 9/9 patients	Hip: 5 lesions, shoulder: 3 lesions, elbow: 5, knee: 5, ankle: 2
Seipel et al.^169^	1 463 total patients, 17–77 years, mean: 40.4 years, 916 M, 547 F 30 NHO patients, 23 M, 7 F Mean time to HO diagnosis: 7.2 ± 1.2 weeks	TBI and SCI TBI/SCI: 23 patients Concomitant peripheral trauma: 5 patients Non traumatic: 7 patients	Hip: 42 lesions, shoulder: 22 lesions, elbow: 7 lesions, knee: 10 lesions, upper ankle joint: 1 lesion, diaphysis of long bones: 3 lesions X-ray
Singh et al.^170^	18 SCI patients, 18–54 years, mean: 32 years, 16 M, 2 F 7 SCI-NHO patients, 18–40 years, mean: 30 years Average abbreviated injury scale (AIS): A Average HO score: 1^a^	SCI	Hip: 7 patients X-ray, SPECT
Wittenberg et al.^171^	413 SCI patients, mean: 35.4 years, 274 M, 82 F 71 SCI-NHO patients, mean: 33.8 years, 63 M, 8 F 30 tetraplegia, 39 paraplegia 57 excluded from study	SCI Concomitant head injuries: 23 patients Concomitant extremity injury: 19 patients Concomitant pelvic injury: 1 patient Concomitant injuries (abdominal and thoracic): 33 patients	Left hip: 70.4%, right hip 57.8% Elbow: 5, knees, 2 X-ray
Bravo-Payno et al.^172^	654 SCI patients 85 SCI-NHO patients, 18–56 years, 29.70 years 41 excluded from study	SCI	Hip: 36 patients, shoulder: 3 patients, elbow: 1 patient, knee: 4 patients X-ray
Orzel and Rudd^173^	50 total patients 43 NHO patients, 18–56 years, 30 M, 13 F	SCI, TBI, peripheral trauma (cerebral vascular insult, burn) SCI trauma: 27 Paraplegics: 17 Closed head injury: 7 Peripheral trauma: 8	Hip: 33 patients, shoulder: 8 patients, thigh: 10 patients, elbow: 8 patients, knee: 3 patients, leg: 1 patient Bone scintigraphy

*F* female, *M* male, *NR* not reported, *TBI* traumatic brain injury, *SCI* spinal cord injury, *NHO* neurological heterotopic ossification, *FX* fracture, *SPECT* single-photon emission computed tomography

^a^HO grade according to Brooker classification

Whether individuals are genetically predisposed towards NHO formation has not yet been established. Initial investigations have examined the association between human leukocyte antigen (HLA) serotypes and NHO; although, there has been contradicting evidence regarding the role of HLA-B27.^17,18^ In a clinical study involving 43 patients with a SCI, five of the 21 patients that developed NHO were positive for HLA-B27 compared to none of the 22 patients without NHO.^17^ However, the hypothesis that HLA-B27 may be a genetic risk factor for NHO was challenged by a separate study that found no differences in the frequency of any HLA-A and HLA-B antigens in 24 NHO patients compared to 740 healthy controls.^18^ Further large-scale studies are required to determine the link between HLA antigens, and other genetically determined factors, that may contribute to NHO.

Several risk factors have been linked to NHO formation, including coma duration, artificial or mechanical ventilation, duration of immobilisation, elevated serum alkaline phosphatase (ALP) levels, and the presence of TBI featuring diffuse axonal injury.^8,19–21^ Patients with TBI-induced NHO often have longer mechanical ventilation and coma duration when compared to TBI patients who do not develop NHO; nevertheless, the exact relationship between coma duration and NHO formation is yet to be established.^22^ It has been proposed that the homoeostatic balance between calcium, oxygen, and pH levels are altered via artificial ventilation, which may result in respiratory alkalosis, contributing to accelerated ectopic bone formation.^19^ However, as increased coma duration is often the result of more severe injuries, it is difficult to determine the effect it has on NHO. These factors may contribute to the increased prevalence of NHO in patients following a stroke, TBI, or SCI.

Recent studies have identified an increased propensity to develop NHO following combat-related trauma, such as blast-TBI (bTBI) and limb amputations.^23^ A recent study on Iraq war operations reported that ~80% of injuries were the result of explosive devices (e.g. improvised explosive devices, mortar, or mines).^24^ Tissue damage caused by bTBI is induced by a combination of shockwaves, supersonic flow and highly heated air flow, resulting in a string of consequences that involve thermal injury, cavitation, and increased intracranial pressure.^25^ An initial study examined the prevalence of NHO/HO during the recent conflict in Iraq and Afghanistan.^26^ Approximately 70% of patients exposed to blast injuries requiring at least one orthopaedic procedure developed HO, while 86% of patients who experienced a bTBI and orthopaedic procedures developed NHO.^26^ Furthermore, univariate analysis demonstrated a significant relationship between HO and TBI severity. These findings demonstrate that the aetiology of polytrauma is incredibly heterogeneous, and as such, NHO may require several therapeutic approaches.^27^

## Pathophysiology of CNS injury

TBI and SCI both induce an array of pathophysiological alterations that may stimulate either formation or resorption of bone.^28,29^ Briefly, acceleration–deceleration and/or rotational forces at the moment of impact can induce significant damage to neurons, glia and the vasculature, triggering a complex cascade of cellular and molecular changes that may contribute to further damage over the ensuing hours, days and months following injury. Common secondary injury mechanisms involved in TBI/SCI can include excitotoxicity, ionic imbalances, mitochondrial dysfunction, oxidative stress, neuroinflammation, ischaemia and edema.^30,31^ Notably, damage to the blood–brain barrier (BBB) or blood spinal cord barrier (BSB), the semi-permeable anatomical interfaces that separate the brain and spinal cord from peripheral blood circulation,^32^ creates potential for abnormal passage of molecules and cells in and out of the brain and spinal cord.^33–36^ For example, substances that are highly concentrated in the CNS (e.g. neuropeptides) or increased following injury (e.g. inflammatory mediators, growth factors) can migrate into the peripheral circulation, and thereby potentially drive NHO formation. This notion is supported by findings that serum and CSF from TBI patients has been found to increase osteoblastic proliferation.^37,38^ Furthermore, prolonged pituitary dysfunction is common after TBI,^39,40^ with alterations to the release of hormones such as parathyroid hormone^41^ and growth hormone,^42^ which may influence musculoskeletal tissues and potentially contribute to NHO development.

## CNS injury may promote bone formation

For quite some time, orthopaedic surgeons have observed that peripheral bone fracture callus formation appears to be significantly enhanced in patients with a TBI. More recently, several clinical studies have supported this anecdotal evidence of increased callus size in TBI patients.^43–45^ However, human studies of this nature are often are confounded by variations in the location, nature and severity of both the bone fracture and TBI. Rodent studies that control for these variables have since demonstrated that TBI increases volume and strength of newly formed bone within the healing callus at acute and sub-acute time-points.^46–51^ This phenomenon may also occur following SCI, with a recent study finding that SCI patients with femoral fracture had increased callus volume and accelerated rate of fracture union when compared to patients with an isolated femoral fracture.^52^

A preliminary study in mice has implicated the involvement of neuronal mechanisms in robust callus formation following TBI.^53^ It was reported that mice that received a fracture contralateral to the site of TBI had increased callus bone volume at 5 days post injury when compared to fracture-only mice, whereas callus bone volume in mice that were given a TBI ipsilateral to the fracture was comparable to fracture-only mice.^53^ In contrast to the aforementioned studies,^46–51^ these differences, however, were not observed at later time-points (i.e. 10 or 14 days post injury).^53^ These findings led the authors to suggest that neuronal mechanisms play a significant role in increasing bone formation acutely following TBI by causing contralateral activation of fracture healing.^53^ It still remains unclear as to how TBI/SCI can alter callus formation; however, it appears likely TBI/SCI-induced NHO share a common mechanism.

It is important to acknowledge that in the absence of a peripheral bone fracture, both TBI and SCI have been associated with reduced bone mineral density in rodents,^54,55^ and humans.^29^ These findings may indicate that cellular and molecular changes due to tissue damage (e.g. inflammation) at a peripheral site is required to stimulate NHO and fracture callus formation. Overall, the mechanisms responsible for the paradoxical effects that CNS injuries have on bone are yet to be elucidated.

## Mechanisms of endochondral HO

The process of ectopic bone formation in trauma induced HO is thought to occur via endochondral (rather than intramembranous) ossification. Although the precise mechanisms are not well characterised, a pool of osteoprogenitor cells (OPCs) residing in skeletal muscle combined with factors that are increased in response to trauma, such as inflammatory cells and molecules, enhanced BMP signalling, and hypoxia are thought to create an environment that together facilitates formation of bone by endochondral ossification.^56,57^ The formation of ectopic endochondral bone begins with invasion of immune cells, including neutrophils, macrophages, mast cells.^2,57^ The influx of inflammatory factors to the often hypoxic and acidic peripheral injury site is thought to stimulate the differentiation of OPCs to fibroblasts, which is driven by expression of fibroblast growth factors (FGFs), which form fibrous tissue.^58–60^ In response to hypoxia, expression of hypoxia-inducible factor-1 alpha (HIF-α) and vascular endothelial growth factor are upregulated to stimulate angiogenesis which provides a conduit for cells to migrate to the injury site.^61^ Moreover, a hypoxic environment induces expression of transcription factor SOX-9, which promotes the differentiation of chondrocytes by activating SOX-9 in a HIF-1α dependent manner.^62^ These chondrocytes then undergo hypertrophy and begin to form a cartilaginous matrix.^63–65^ Subsequent remodelling of this cartilage is mediated by matrix metalloproteinases and results in the release of angiogenic factors that further promote vascular invasion.^66^ The cartilage is then removed as the lesion begins to mineralise. Over time the initial woven bone is then remodelled to form mature lamellar bone with a marrow cavity. Of interest, sources of OPCs have not yet been established, however it has been suggested that that induction of OPCs from muscle satellite cells cause HO formation.^19,67,68^ Other studies suggest that the source of OPCs are from fibroadipogenic progenitors that reside within the muscle interstitium, but are not exclusive to muscle.^69^ It is possible that identification of the exact source of these OPCs may facilitate the design of better therapeutic strategies to prevent HO formation. There is little information regarding whether the mechanisms of NHO differ from HO, but it is important to establish as it has ramifications for identifying potential druggable targets for each condition. For example, although peripheral trauma associated HO is thought to occur exclusively via endochondral ossification, it is not yet known whether the additional presence of neurotrauma may alter the frequency of endochondral vs. intramembranous ossification. Additionally, an initial human study provides evidence that the histological mechanisms of ectopic bone formation were identical in lesions from TBI, SCI and trauma induced HO.^70^ However, significant heterogeneity existed between the location of HO, furthermore the time-point that the HO was excised was not stated. Therefore, it is difficult to determine whether the mechanisms differ between HO and NHO in the acute phase, (i.e. before mineralisation). For a more in-depth description of the cellular and molecular mechanisms of HO formation, the reader is referred to the following reviews.^71–75^

## Mechanisms of HO formation following CNS injury

**Fig. 1:**
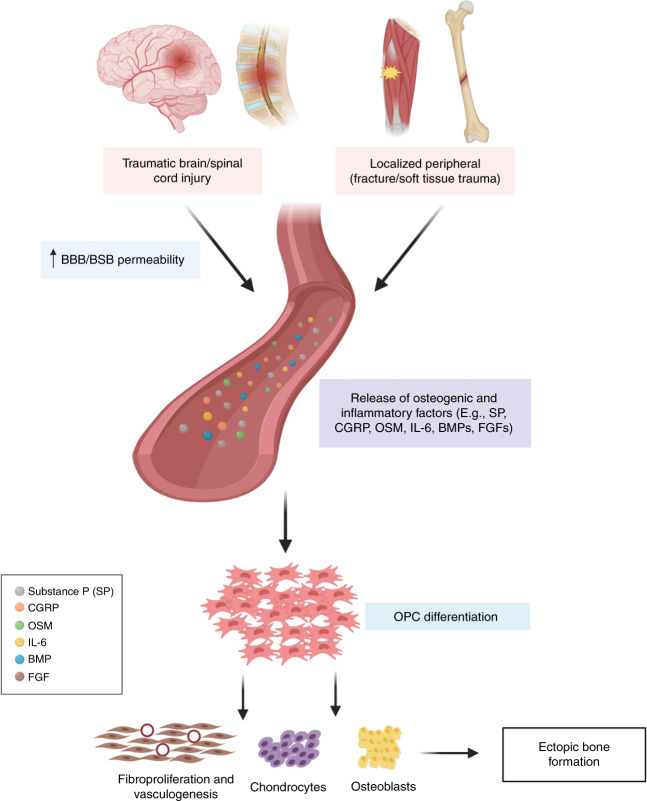
Proposed mechanism for NHO development. Simultaneous injury to the CNS and peripheral sites triggers the release of osteogenic and inflammatory factors including; SP, CGRP, OSM, IL-6, BMPs and FGFs. The influx of osteogenic and inflammatory factors, initiates the differentiation of OPCs into fibroblasts which is mediated by fibroblast growth factors (FGFs). This influx also elicits angiogenesis, which results in an increase in oxygen tension, triggering the differentiation of OPCs into chondrocytes which undergo hypertrophy and form a cartilage matrix. This cartilaginous matrix provides a structural framework for the formation of blood vessels, osteoblast proliferation and differentiation and formation of ectopic bone (created with BioRender.com)

Current understanding of the cellular and molecular mechanisms of HO formation specifically in the context of neurotrauma is lacking; however, there are a number of potential mechanisms through which CNS injury may promote formation of ectopic bone at peripheral injury sites (summarised in Fig. 1). Indeed, the past decade has seen the emergence of animal models of polytrauma that have shed some light on the key drivers of HO formation following TBI or SCI. Herein, we outline emerging evidence and hypotheses of the mechanisms of NHO formation, with a particular focus on the potential role of macrophages and neuropeptides.

### Role of inflammatory cells and mediators

#### *Macrophages and neutrophils*

Macrophages and neutrophils have been identified as prominent cells of the muscle inflammatory infiltrate in the acute and sub-acute phases post injury and may contribute to NHO. Work by Genet et al. has highlighted the contribution of F4/80^+^ resident tissue macrophages as key drivers of SCI-induced NHO.^12^ Following SCI and cardiotoxin induced muscle injury, mice were injected intravenously with clodronate-loaded liposomes to deplete resident tissue macrophages.^12^ Ablation of these resident tissue macrophages was found to reduce NHO volume by ~90%, and completely prevent NHO development in 3/11 mice.^12^ These findings indicate that macrophages likely play a prominent role in SCI-induced NHO formation. Resident tissue macrophages have previously been implicated in the development of HO, however it still remains unclear as to whether the contribution of resident tissue macrophages differs between HO and NHO. To the best of our knowledge, to date no studies have compared the effect of resident tissue macrophage depletion in HO and NHO models. However, in a model of HO induced by burn and tenotomy, although injection of mice with clodronate-loaded liposomes decreased total HO volume by ~50%, HO was still observed in all mice.^76^ Although further studies are required, taken together these findings suggest that the contribution of resident tissue macrophages may differ between HO and NHO.

A number of factors released by macrophages are also released by neutrophils, therefore to determine the role of neutrophils in SCI-induced NHO, Tseng et al. recently examined NHO formation in neutropenic mice.^77^ Neutrophil depletion had no effect on NHO volumes.^77^ Nor did treatment with rhG-CSF which significantly increased the number of neutrophils in the blood, bone marrow and injured muscles.^77^ These findings suggest that macrophage related factors in combination with CNS injury drive NHO.

#### *OncostatinM*

Oncostatin M (OSM) is an inflammatory cytokine, derived from activated macrophages, osteoclasts, monocytes, T cells, and neutrophils.^78,79^ OSM has been reported to stimulate osteoblastic differentiation and hence bone formation by acting on osteoclasts and osteoprogenitors.^80^ The involvement of OSM in osteogenic differentiation in NHO development suggests that OSM receptor (OSMR) and OSM could be viable therapeutic targets.^78^

A recent study examined NHO formation using NHO-lesions from 64 patients with CNS injuries (SCI, TBI, stroke, or cerebral anoxia) and a mouse model of SCI-induced NHO.^78^ Histological analysis of NHO-lesions excised from CNS-injured patients revealed that OSM was expressed by CD68^+^ macrophages and osteoclasts within NHO sections.^78^ Of note, OSM plasma protein levels were elevated twofold when compared to healthy donors (i.e. HO negative patients following total hip surgery), suggesting that plasma OSM levels may serve as a biomarker of NHO formation.^78^ It was also found that muscle-derived stromal cells isolated from NHO-lesions expressed OSMRs, and that treatment with recombinant human OSM increased mineralisation and differentiation.^78^ In addition, after SCI-induced NHO in mice, immunohistochemistry and mRNA analysis demonstrated that OSM levels were significantly increased in injured muscle post-CDTX injection and SCI, and that OSM is secreted and accumulates at the site of NHO.^78^ Furthermore, deletion of the OSMR receptor significantly reduced NHO volume (median volume 14.2 mm^3^ in wild type, 3.2 mm^3^ in OSMR knockouts). Considered together, these findings suggest that OSM produced by resident tissue macrophages drives NHO formation by stimulating differentiation and mineralisation of muscle stromal cells and that OSM may represent a plasma marker and therapeutic target for preventing/reducing NHO formation.^78^ The contribution of OSM to TBI-induced NHO is yet to be elucidated in a suitable model, as an animal model of TBI-induced NHO that accurately mimics the combinations of injuries that these patients often present with has only recently been developed.^81^ Briefly, this model features a concomitant femoral muscle crush injury, femoral fracture and a moderate-severe brain injury in rats, where 70% of rats that underwent these injuries developed ectopic bone at the peripheral injury site.^81^

### Role of neuropeptides

Neuronal injury can trigger neurogenic inflammation, which in the context of moderate-severe TBI or SCI has been shown to exacerbate secondary injury pathologies such as neuronal cell death,^82^ BBB^83^ and BSB,^82^ oedema,^83^ ischaemia^83^ and hypoxia.^84^ Neurogenic inflammation has been associated with the release of neuropeptides, particularly substance P (SP) and calcitonin gene related protein (CGRP) which increases vascular permeability and vasodilation respectively.^85^ Impairment of the BBB following trauma can promote further propagation of neurogenic inflammatory factors, causing exacerbated neural injury.^86^ Notably, there is now emerging evidence that release of these neuropeptides into peripheral circulation after neurotrauma may be a key driver of NHO formation.

#### *SubstanceP*

SP is a neuropeptide that is distributed throughout the central and peripheral nervous system, with increasing evidence highlighting its role in neurogenic inflammation, bone remodelling and TBI pathology.^87,88^ SP has previously been identified as a potential therapeutic target that contributes to NHO and HO development.^12,89,90^ SP possesses a strong affinity to neurokinin-1 receptor (NK-_1_R) belonging to the tachykinin receptor group.^91^ Accumulating evidence indicates that SP contributes to NHO development. For instance, several human and animal studies have reported that SP levels are elevated in the blood following TBI and SCI.^92,93^ Most notably, the role of SP in NHO has been studied in patients a murine model of SCI-induced NHO.^12^ SP concentrations were significantly higher in plasma from NHO patients compared to healthy volunteers. While in the mouse model, antagonising SP receptor NK-_1_R with RP67580 reduced NHO volume by ~30%.^12^ These findings indicate that SP may represent both a prognostic biomarker of NHO and a treatment target, whereby an intervention that downregulates SP is initiated following elevated plasma levels of SP.

Mast cell degranulation has been reported to be essential for SP to induce HO formation.^94,95^ Further, mast cells also release serotonin, which is known to have dual functions in bone remodelling dependent upon the site of production and it has been proposed that serotonin may drive adipocyte differentiation, creating a further hypoxic microenvironment for NHO formation.^96^ With respect to TBI-induced NHO, the role of SP has yet to be examined. However, the release of SP following TBI is associated with increased BBB permeability, brain oedema formation, as well as increased intracranial pressure, which contributes to neuronal cell death after the initial trauma.^97^ Therefore, it is likely that targeting SP may affect NHO either acting locally by preventing ectopic bone formation at the peripheral injury site or by acting centrally where attenuating TBI outcomes may result in reduced NHO volume.

Altogether, these findings propose that SP does play a significant role in both NHO and HO formation. Given the essential role of SP in acute CNS injuries, further studies need to be carried out to potentially use SP as a therapeutic target and blood-based biomarker of NHO.^98^

#### *Calcitonin gene related protein (CGRP)*

CGRP is a sensory neuropeptide that is distributed in both the CNS^99^ and PNS.^100^ In the CNS, CGRP is expressed in cerebral cortex, hippocampus and hypothalamus,^99^ while in the CNS^99^ and PNS^100^ can be found in sensory, motor neurons and often colocalizes with SP.^100^ The upregulation of CGRP following TBI is also thought to contribute to neurogenic inflammation.^85^ While the precise role of CGRP in the development of NHO remains to be elucidated, in a mouse model of SCI-induced NHO significantly elevated levels of CGRP were found within the injured muscle 14 days post injury.^101^ In vitro, CGRP was found to promote the differentiation of fibroadipogenic progenitor cells to chondrocytes.^101^ Interestingly, studies in rodents have revealed that TBI,^102–104^ or SCI^104^ concomitant with fracture elevates CGRP serum levels, with these animals having accelerated bone healing. This suggests that increased expression of CGRP following CNS trauma and peripheral injury may contribute to heterotopic bone formation by triggering neurogenic inflammation; however, further studies examining the relationship between SP, CGRP and NHO are required to determine its exact mechanism.

#### *Other neuropeptides*

Neurotrophins such as nerve growth factor (NGF), neurotrophin-3 (NT-3), neutrophin-4 (NT-4), and brain-derived neurotrophic factor have all been associated with alterations in bone metabolism. NGF is responsible for the growth and maintenance of neuronal and non-neuronal cells in both PNS and CNS.^105^ However, a growing body of evidence suggests that NGF and NT-3 may play a role in skeletal development,^106^ fracture healing^107–109^ and HO.^110^ For example, in a rat model of HO which features bilateral midpoint Achilles tenotomy, mRNA expression of NT-3 was significantly (100-fold), while NGF levels were more modestly increased (<20-fold) from 4 weeks to 12-week post-tenotomy.^110^ The role that these neuropeptides play in NHO is yet to be reported in the literature.

### Disrupted neural signalling

There is increasing recognition that bone modelling and remodelling can be regulated by the CNS, with hypothalamic leptin signalling being a key regulator of bone remodelling.^111^ Although the precise mechanisms are unclear, it has been theorised that central regulation of bone formation occurs via activation of efferent pathways relayed via the brainstem.^111,112^ As such, damage or alterations in excitability of these neural pathways following TBI or SCI may also be a contributor to NHO formation. Supporting this hypothesis, ventromedial hypothalamic neurons have been identified as playing a key role in bone formation, with chemical lesioning of these neurons resulting in a high bone mass phenotype in mice.^113^ This was thought to occur via ablation of leptin receptors which are densely populated in this region, thus inhibiting the osteogenic effect of leptin.^113^ Indeed, future studies are required to elucidate the role of efferent signalling on NHO, as well as the effect that lesions to different structures of the brain have on TBI-induced NHO.

## Treatments for NHO

**Fig. 2:**
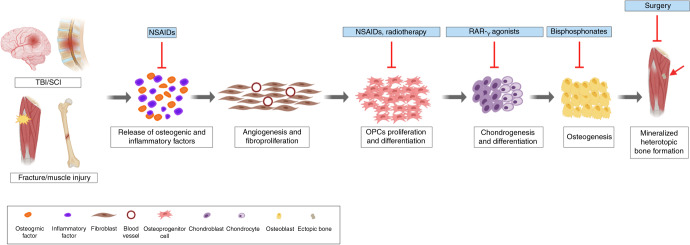
Current treatments targeting specific pathways of NHO. NHO development is triggered by a cascade of inflammatory factors. Presently, the preferred prophylactic treatment for NHO/HO involves NSAIDs (e.g. Indomethacin) to downregulate the inflammatory response and prevent OPC differentiation. Radiotherapy is thought to prevent the formation and development of ectopic bone specifically by inhibiting the differentiation of OPCs. RAR-_γ_ agonists have been shown to prevent chondrogenesis and therefore subsequent mineralisation. While, nitrogen-containing bisphosphonates (e.g. sodium etidronate) have been used to inhibit mineralisation, and the formation of ectopic bone. Finally, when bone is completely mineralised, surgical resection is the only remaining intervention. This invasive procedure, however, is accompanied by the risk of recurrence and is associated with complications which include incomplete resection, functional and physiological impairment (created with BioRender.com)

In this section, an overview of existing therapeutic interventions for NHO will be provided. These approaches are summarised in Fig. 2. These strategies include surgical resection of completely mineralised ectopic bone, radiotherapy, non-steroidal anti-inflammatory drugs (NSAIDs) and bisphosphonates. In addition, important considerations for these treatments in the context of polytrauma involving CNS injury are discussed.

### Surgical excision

Presently, invasive surgical resection is the only effective clinical approach to cure NHO.^6,7^ However, it is recommended that surgery should only be considered if NHO patients fulfil the following criteria: (1) a significant reduction in range of motion (ROM) due to joint ankylosis, (2) an absence of acute inflammatory response and (3) the lesion is sufficiently mineralised (mature) to enable excision.^1,114,115^ However, several other factors are also to be considered when deciding the timing of surgical intervention. Previous studies have found that to reduce the risk of recurrence, surgical excision is preferred after ectopic bone has fully mineralised.^10,116,117^ Following SCI and TBI, resection was traditionally performed >12–18 months-post injury.^1,118^ Over the past decade there has been a shift in the clinical management of NHO to favour earlier resection i.e. surgery is performed as soon as the patient is stable enough to undergo surgery and the lesion is sufficiently mineralised to enable resection.^8,119–121^ These changes were based on findings that earlier resection of NHO-lesions did not in fact increase the risk of recurrence.^8,119–121^ Further, recent evidence suggests that early excision may reduce the risk of operative complications (e.g. peri-operative fracture), enhance bone and articular cartilage health, and reduce negative cerebral changes (e.g. atrophy of motor areas) that further inhibit ROM (see Table 2).^11,118,122^

**Table 2:** Treatments for clinical and radiographical evidence of HO/NHO reported across literature

Author	Description (*total number of* patients, age range, mean age, gender, *number of* patients with NHO)	Injury and imaging modality used to confirm NHO	Location of NHO and treatment/s	Reported recurrence and complications
Surgical resection				
Meiners et al.^174^	29 SCI-NHO patients, 27–68.13 years, mean: 37.87 years, 28 M, 1 F 41 lesions Cervical lesions of spinal cord: 10 patients Thoracic spine lesions: 19 patients	SCI X-ray	Hip Dose: average: 9.17 Gy, range: 0.7–12 Gy in 1–5 sessions Mean follow-up: 4.2 years Mean time to surgery: 82.1 months (17–298 months) Indications for surgery: seating problems, loss of functions, pressure sore, pain Preoperative ROM: 21.95° (range: 0–80°) Postoperative ROM: 82.68° (range: 0–120°)	Recurrence: 3 patients Complications: deep and superficial wound infections, fracture, aneurysm and pressure ulcer
Hunt et al.^175^	42 burns patients, 22–62 years, mean: 38 years 42 burn-HO patients, 22–62 years, mean: 38 years 47 lesions Mean TBSA: 55% Mean third degree burn: 37% Average ventilator support: 58 days	Burn injuries X-ray	Hip, elbow, forearm Indications for surgery: decreased ROM resulting in loss of functions in daily activities, ulnar nerve entrapment, inability to perform physical therapy Preoperative ROM: 52° Postoperative ROM: 119°	Recurrence in 6 elbows, 1 hip and 1 forearm Complications: ulnar nerve deficit, numbness weakness, small haematoma, minor wound dehiscence and cellulitis.
Radiation therapy				
Hamid et al.^134^	45 patients with elbow trauma, 18–65 years, mean: 44 years, 25 M, 20 F 20 elbow trauma-HO patients	Intraarticular distal humeral fracture, Fracture-dislocation with proximal radial and/or ulnar fracture X-ray, CT scan	Elbow Dose: 700 cGy single fraction dose at 6-MeV photons), *N* = *21* Mean follow-up: 7.5 months (range: 6–26 months) Mean time to treatment: 72 h Indications for treatment: seating problems, loss of functions, decubitus, pressure sore, pain Preoperative ROM: 21.95° (range: 0–80°) Postoperative ROM: 82.68° (range: 0–120°)	Trial was terminated early due to high non-union rate observed in the radiation treatment group Recurrence: 0 Complications: infection (2), non-union (8)
Stein et al.^176^	11 patients with elbow trauma, 28–78 years, mean: 51 years, 3 M, 8 F 3 elbow trauma-HO patients, 54–78, mean: 63 years, 1 M, 2 F 3 lesions	Fracture/dislocation of the elbow Radiographs	11 patients Dose: 700 cGy single non-fractionated at unreported MeV Mean follow-up: 12 months (range: 9–24 months) Mean time to treatment: 5 days (range: 0–16 days) Indications for treatment: NR Preoperative ROM: NR Postoperative ROM: 114.5° (range: 0–135°)	Recurrence: 0 Complications: decreased sensation along ulnar nerve
Müseler et al.^177^	244 SCI-NHO patients, 18–81 years, mean: 46.4 years, 207 M, 37 F, 444 lesions AIS A—12 patients (4 tetraplegic 8 paraplegic) AIS B—1 patient (1 tetraplegic)	SCI CT scan or MRI	Radiation therapy (7 Gy, single dose accompanied by 15MV or 6MV) Mean follow-up: 89.4 days Mean time to treatment: 3.7 days Indications for treatment: NR	Recurrence: 13 patients (26 joints) Complications: NR
Cipriano et al.^178^	60 NHO-patients, mean: 36.7 years, 47 M, 13 F 72 lesions	TBI, SCI, TBI + SCI, TBI + local trauma	30 patients Dose: 700 cGy dose of radiation Mean follow-up: 12.7 months (range: 6–33 months) Mean time to treatment: 1.18 days (range: 0–4 days) Indications for treatment: limited ROM, nerve impingement, reduced quality of life and functions Preoperative ROM: Postoperative ROM: Hip—4.23°, Hip—67.2° Knees—81.3°, Knees—117.5° Elbows—4.0°, Elbows—140.0°	Recurrence: 6 joints Complications: NR
NSAIDs				
Banovac et al.^138^	33 SCI-NHO patients AIS A—13 (5 tetraplegics, 7 paraplegic) AIS B—1 (1 tetraplegic) AIS C—2 (2 paraplegic) AIS D—1 (1 tetraplegic)	SCI Bone scintigraphy (early stage) X-ray (later stage)	16 patients Oral indomethacin 75 mg daily, IV disodium etidronate, 300 mg daily for 3 days, oral etidronate, 20 mg·kg^−1^ per day for 6 months Mean follow-up: 1.5 months Mean time to treatment: 21 days Indications for treatment: local erythema, swelling, loss of joint ROM and fever	Recurrence in 2 patients Complications: upper abdominal discomfort
Banovac et al.^179^	76 SCI patients, 65 M, 11 F AIS A—28 patients AIS B—8 patients AIS C—1 patient	SCI Bone scintigraphy, radiograph	37 patients Oral rofecoxib 25 mg daily, IV disodium etidronate 300 mg daily for 3 days, oral etidronate, 20 mg·kg^−1^ per day for 6 months Mean time to treatment: 25 days Indications for treatment: local oedema, fever and decreased joint ROM	Recurrence in 5/37 patients Complications: NR
Romano et al.^180^	400 THA patients, mean: 61.2 years 24 excluded (due to side effects)	Coxarthrosis, femoral head necrosis Radiograph	250 patients Rectal indomethacin 50 mg daily for 2 days, a day post-surgery followed by oral indomethacin 50 mg daily for 18 days 150 patients Celecoxib 200 mg daily for 2 days, starting 2 days post-surgery for 20 days Mean follow-up: 12 months Mean time to treatment: 1 and 2 days respectively	Indomethacin: 40 patients, Celecoxib: 21 patients Complications: (Indomethacin) gastrointestinal side effects, excessive bleeding, mental confusion (Celecoxib), nausea and gastrointestinal pyrosis
Schmidt et al.^181^	201 THA patients, 28–89, mean: 67.5 years	Total hip replacement Radiograph	102 patients Oral indomethacin 25 mg, thrice daily, for 6 weeks, starting on first postoperative day Mean follow-up: 12 days Mean time to treatment: 1 day	Recurrence in 13 patients Complications: NR
Bedi et al.^182^	616 patients after hip arthroscopy, mean: 31.3 years, 342 M, 274 F 29 HO patients, 15–57 years, mean: 30.6 years, 21 M, 8 F	Hip arthroscopy Radiographs, CT scan	277 patients Naproxen (500 mg, twice daily for 30 days, starting a day post-surgery) 339 patients Indomethacin 75 mg daily for 4 days, Naproxen 500 mg, twice daily for 30 days Mean follow-up: 13.2 months (range: 2.9–16.5 months) Mean time to treatment: 1 day 7 patients HO surgical excision, radiation therapy 700 cGy, single dose Mean time to treatment: 11.6 months (range: 5.2–16.2 months)	*Naproxen only:* 23 patients have HO Naproxen + Indomethacin: 6 patients Complications: NR
Beckmann et al.^183^	106 patients after hip arthroscopy, mean: 35 years, 40 M, 66 F Excluded from study: *n* = 10	Hip arthroscopy Radiographs	52 patients Naproxen 500 mg, twice daily for 3 weeks, post-surgery Mean time to treatment: 1 day Indications for treatment: pain, radiographic abnormalities and evidence of labral tear on MRI	Recurrence: 2 Complications: Gastrointestinal discomfort, rash, blood clot, heartburn, headache and pain
Neal et al.^184^	2 649 THA patients, mean: 65.5 years, 1 311 M, 1 338 F 601 excluded 627 lesions	Hip arthroplasty Radiograph	1 039 patients Aspirin 162 mg·d^−1^ for 35 days post-surgery Mean follow-up: 22 months	Recurrence: 627 patients Complications: hip pain (with the need for analgesia), difficulty or restriction of mobility
Bisphosphonates				
Schuetz et al.^185^	7 patients in total, 47–68 years, mean: 54.8 years, 7 M 5 patients with HO, 47–68 years, mean: 54.8 years, 5 M Number of lesions: 8	Caisson disease, tetraplegia, e.coli sepsis, osteoarthritis, FOP Radiographs	IV pamidronate 680 mg/850 mg/1200 mg Mean follow-up: 19.6 months (range: 4–54 months) Indications for treatment: pain, hardening at operation site and decreased ROM	Recurrence in 1 patient Complications: need for pain medication, lower back pain
Orzel and Rudd^173^	50 patients 43 NHO patients, 18–56 years, 30 M, 13 F 81 lesions	SCI paraplegia, closed head injury, peripheral trauma, cerebral vascular insult, burn Bone scintigraphy	14 patients Oral etidronate disodium 20 mg·kg^−1^ for first 2 weeks followed by 10 mg·kg^−1^ for remainder of study Mean follow-up: 22.5 months Indications for treatment: Radiograph evidence	No response to therapy in 4/14 Complications: NR
Banovac^154^	40 SCI-NHO patients, mean: 23 years, 39 M, 1 F AIS A—37 patients (16 are tetraplegic, 21 are paraplegic) AIS B—3 patients (2 are tetraplegic, 1 is paraplegic)	SCI Radiograph and bone scintigraphy	40 patients IV etidronate sodium 300 mg, 3 doses for 3 days followed by oral etidronate sodium 20 mg·kg^−1^ per day for 6 months Mean follow-up: 35 months Indications for treatment: oedema, reduced ROM, fever, positive scintigraphy	Recurrence in 2 patients, Complications: NR
Banovac et al.^152^	27 SCI patients, 16–54 years, mean: 36 years, 25 M, 2 F 11 SCI-NHO patients 3 excluded	SCI Bone scintigraphy	24 patients IV etidronate disodium (300 mg for 3 h, 3 doses for 3 days/5 days) followed by oral etidronate sodium (20 mg·kg^−1^ per day for 6 months) Indications for treatment: acute swelling, reduced ROM, increased body temperature, laboratory test (increased serum alkaline phosphatase, accelerated erythrocyte sedimentation rate) and positive bone scintigraphy	Recurrence: 11 patients
Garland et al.^151^	75 SCI-patients 14 SCI-NHO patients, 17–30 years, mean: 25 years 5 excluded 14 lesions	SCI Radiograph, bone scintigraphy	9 patients Sodium etidronate 20 mg·kg^−1^ per day for 2 weeks followed by 10 mg·kg^−1^ per day for 2 years Mean follow-up: 14 months (range: 5–19 months) Mean time to treatment: 26.7 days (range: 0–55 days) Indications for treatment: swelling, reduced ROM	Recurrence: none Complications: none

*F* female, *M* male, *NR* not reported, *TBI* traumatic brain injury, *SCI* spinal cord injury, *NHO* neurological heterotopic ossification, *ROM* range of motion, *TBSA* total body surface area (%), *THA* total hip arthroplasty, *FOP* fibrodysplasia ossificans progressive

Resection has however been associated with a number of complications. For example, complete excision of periarticular NHO is particularly difficult, with patients often left with persistent decreases in ROM. Lesion remnants can result in both functional and physiological impairment due to impingement of neurovascular bundles, ankylosis, and pain.^10,118,123–125^ Like any other invasive procedures, post- and intra-operative NHO excision is associated with potential blood loss and infection—both complications that may have a substantial effect on TBI or SCI recovery. Moreover, surgical removal can damage adjacent peripheral tissues, and recurrence at the site of excision is common.^123,126–129^ As such, surgical intervention is not an optimal NHO therapy and should be carefully considered.

### Radiation therapy

Radiation therapy is thought to prevent the formation and/or progression of HO by inhibiting the differentiation of OPCs.^114^ Specifically, in vitro studies have demonstrated that radiotherapy inhibits BMP-2 signalling, reduces osteoblastic proliferation and differentiation, and promotes apoptosis.^130,131^ In an initial pre-clinical study, adult rats implanted with de-mineralised bone matrix were administered radiation at 2-, 4-, 6-, 8-, 10- and 12 days post-implantation.^132^ Implanted rats went on to form ectopic bone at 11 days post-implantation.^132^ It was noted that rats that underwent radiation at 2- or 4 days post-implantation had reduced HO volume by ~60% and 24% respectively. However, when radiation was delayed until 8 days post-implantation, the authors observed no difference in HO volume between rats that were irradiated and controls.^132^ Several studies have reported beneficial effects of administering radiotherapy to prevent NHO post-TBI and SCI, or to prevent the recurrence of NHO (i.e. post-excision; see Table 2).^133–135^ For example, in a phase I/II clinical study, 33 SCI patients that underwent radiotherapy observed no further ectopic bone growth, however joint mobility was mildly affected in three patients.^133^ In some cases, the risk of impaired fracture healing can be prevented in radiotherapy by adequately shielding the surrounding areas of interest; however, this can be difficult when ectopic bone forms close to fractures and around amputation sites. In one particular clinical study where patients with elbow injuries underwent radiation therapy, eight of the 21 patients experienced fracture non-union, whereas for the 24 patients that did not undergo radiotherapy, only one experienced non-union.^134^ In addition to fracture healing, radiation therapy can also disrupt wound healing, and has been associated with an increased risk of malignancy.^136^ Therefore, the use of radiotherapy in polytrauma NHO patients is often contraindicated.^137^

### Non-steroidal anti-inflammatory drugs (NSAIDs)

NSAIDs have been successfully used to prevent ectopic bone formation following SCI and hip arthroplasty (see Table 2). While NSAIDs such as celecoxib and meloxicam have been used to prevent NHO, indomethacin, a non-selective COX-1 and COX-2 inhibitor, is currently considered the gold standard for preventing NHO formation and progression.^118,138^ The effect of indomethacin has been demonstrated in a rat model of HO that features subcutaneous implantation of de-mineralised bone matrix.^139^ When indomethacin was administered 6 h prior to the implantation, there was a reduction in area of ectopic bone, ALP activity, and calcium content when compared to controls.^139^ However, when indomethacin was administered at the time of de-mineralised matrix implantation or post-implantation (6 h, 1 d, 2 d, 3 d, and 4 d), there were no differences in ectopic bone area, ALP activity, and calcium content when compared to controls.^139^

Despite the proven efficacy of NSAIDs for treating NHO, it is important to recognise that there are reports of potentially negative adverse effects of these agents on patients with polytrauma. For example, rofecoxib, a highly selective COX-2 inhibitor was frequently prescribed to prevent HO.^140^ However, it was withdrawn from the market following a randomised, placebo-controlled, double-blind clinical trial that found chronic use elevated the risk of serious cardiovascular events (i.e. heart attack and stroke) in patients taking it to prevent the recurrence of colorectal polyps.^140^ Furthermore, evidence suggests that indomethacin may interfere with fracture healing.^141,142^ A study in rats reported that indomethacin treatment diminished mechanical properties of femoral fracture calluses.^141^ This finding suggests that indomethacin treatment is problematic, particularly in patients with concomitant fracture. In addition, ibuprofen, a commonly prescribed NSAID, may worsen cognitive outcome after severe TBI in rats.^143^ Further, rats given a TBI and administered celecoxib, a COX-2 inhibitor often administered to HO patients, had worse motor performance.^144^ In some circumstances COX-1 inhibitors have been associated with an increased prevalence of gastrointestinal side effects such as bleeding and perforations.^145,146^ NSAIDs also have a limited therapeutic window and are only effective in the early stages of HO development, prior to the formation of ectopic bone (Fig. 2). Once bone deposition has occurred, NSAIDs are ineffective, hence surgical intervention remains the only option. Overall, despite the efficacy of NSAIDs in preventing/reducing ectopic bone formation, these findings support the notion that careful consideration must be taken before administering NSAIDs to polytrauma patients.

### Bisphosphonates

Bisphosphonates are commonly used to treat bone disorders such as osteopenia, osteoporosis, and Paget’s disease by reducing osteoclastic bone resorption.^147^ Nitrogen-containing bisphosphonates, such as risedronate, sodium etidronate, and alendronate, are often prescribed as prophylaxis due to their ability to effectively prevent mineralisation.^148^ In vitro, treatment of osteoblasts with nitrogen-containing bisphosphonates, pamidronate and alendronate, at doses of 10^−4^–10^−5^ mol·L^−1^ (therapeutic doses range in humans is between 10^−5^ and 10^−9^ mol·L^−1^) results in osteoblastic apoptosis directly, and/or indirectly via osteoblast cell cycle arrest and cell proliferation inhibition.^149,150^ These findings were not observed when cells were treated with non-nitrogen-containing bisphosphonates.^150^ The use of nitrogen-containing etidronate in ectopic bone formation has been well documented.^151–154^ However, some studies do not recommend sodium etidronate as treatment for NHO.^151,153^ As bisphosphonates suppress bone resorption and accumulate in the body for an extended period of time, adverse effects associated with high doses include amassed bone microdamage which is frequently observed with old age, hence further contributing to increased skeletal fragility.^155,156^ In addition, due to the potentially negative impacts on skeletal fragility combined with the high cost of bisphosphonates, it would be beneficial to identify those patients who are at risk of developing NHO, and treat only them with bisphosphonates.^157^ It is also unknown how bisphosphonates affect TBI and SCI outcomes, which should be considered in NHO patients.

### Retinoic acid receptor agonist

**Fig. 3:**
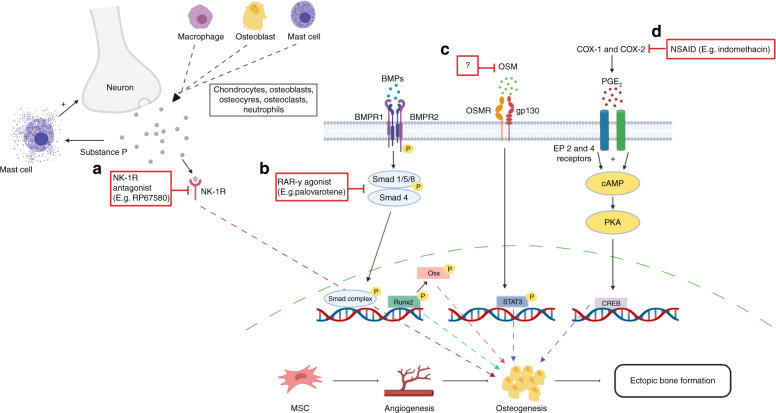
Key pathways/mechanisms implicated in the development of NHO highlighting potential and existing therapeutic targets to mitigate NHO. **a** Substance P receptor (NK-_1_R) antagonists (e.g. RP67580) have been found to reduce NHO volume in murine models. **b** Downstream of the BMP pathway, RAR-_γ_ agonists such as palovarotene have been reported to prevent early stages of NHO development by disrupting OPC differentiation, chondrogenesis and osteogenesis by downregulating mRNA expression of SOX-9 and RUNX2. **c** OSM is a potential therapeutic target and may serve as a biomarker for NHO. Blocking OSM has been found to reduce NHO likely by inhibiting downstream transcription factor, STAT3, which is known to trigger bone formation. **d** NSAID, indomethacin is currently the preferred prophylaxis for NHO/HO. It targets COX-1 and COX-2 non-selectively, inhibiting the production of prostaglandins and osteogenesis (created with BioRender.com)

Retinoic acid receptors (RAR) are mediators of skeletal development via the Smad complex, that plays an integral role in chondrogenesis.^158^ An RAR-_γ_ agonist, palovarotene, has shown to be effective in preventing the initial stages of NHO.^159^ The authors developed a model that mimics the injury combinations and bioburden that occurs in blast related combat injuries.^159^ Palovarotene significantly decreased NHO by inhibiting the expansion as well as differentiation of OPCs into chondrocytes.^159^ Further, palovarotene treatment was found to downregulate mRNA expression of chondrocytic (SOX-9 and collagen2α1) and osteoblastic (OC, OPN, BMP-2, BMP-4, POU5FL and RUNX2) genes Fig. 3.^159–161^

RAR-_γ_ activation, however has been shown to delay growth plate development. Therefore, several studies have warned that precautions should be taken when administering an RAR-_γ_ agonist to children.^160,162^ Whereas in adults, it was proposed that RAR-_γ_ treatment could be given intermittently, henceforth providing sufficient recovery time for the growth plate.^160,162^ Furthermore, as palovarotene has also been shown to inhibit fracture healing care should be taken when administering to patients with healing fractures.^162–164^ Nonetheless, activation of RAR-_γ_ still shows promising results as a novel therapeutic agent in preventing progression of NHO.

## Conclusion

The development of NHO is relatively common after TBI and SCI, and typically occurs in the presence of concomitant significant peripheral musculoskeletal injuries. The majority of NHO cases are diagnosed following extensive mineralisation, at which stage patients are likely to experience considerable pain and disruption to daily functional activities. Current treatments are limited in effectiveness and not always suitable for NHO patients, and there are no reliable prognostic biomarkers to identify patients at high risk of developing NHO to guide preventative interventions. Fortunately, there are numerous promising avenues for future research to identify new underlying pathophysiological mechanisms related to NHO, prognostic biomarkers, and prophylactic therapies that are suitable for complex trauma patients with CNS injuries. These future studies would benefit from a complementary translational approach that incorporates improved clinically relevant animal models in parallel with more rigorous clinical investigations. In doing so, there is the strong potential to develop biomarkers and prophylactic strategies to improve NHO patient outcomes.
